# Self-reported adverse drug reactions and their influence on highly active antiretroviral therapy in HIV infected patients: a cross sectional study

**DOI:** 10.1186/2050-6511-15-32

**Published:** 2014-06-23

**Authors:** Wondmagegn Tamiru Tadesse, Alemayehu Berhane Mekonnen, Wubshet Hailu Tesfaye, Yidnekachew Tamiru Tadesse

**Affiliations:** 1 Department of Pharmacology, School of Medicine, Addis Ababa Science & Technology University, Addis Ababa, Ethiopia; 2 Department of Clinical Pharmacy, School of Pharmacy, University of Gondar, Gondar, Ethiopia; 3 Department of Internal Medicine, School of Medicine, University of Gondar, Gondar, Ethiopia

**Keywords:** Perceived adverse effect, Antiretroviral therapy, Self-report ADRs

## Abstract

**Background:**

Patients on antiretroviral therapy have higher risk of developing adverse drug reactions (ADRs). The impact of ADRs on treatment adherence, treatment outcomes and future treatment options is quiet considerable. Thus, the purpose of this study was to describe the common self-reported ADRs and their impact on antiretroviral treatment.

**Methods:**

Cross-sectional study was conducted at antiretroviral therapy (ART) clinic of Gondar University Hospital. Semi-structured interview questionnaire was used to extract self-reported ADRs, socio-demographic, and psycho-social variables. Variables related to antiretroviral medication, laboratory values and treatment changes were obtained from medical charts. Chi-square and odds ratio with 95% confidence interval were used to determine the associations of dependent variables.

**Result:**

A total of 384 participants were enrolled. At least one adverse drug reaction was reported by 345 (89.8%) study participants and the mean number of ADRs reported was 3.7 (±0.2). The most frequently reported ADRs were nausea (56.5%) and headache (54.9%). About 114 (31.0%) participants considered antiretroviral therapy to be unsuccessful if ADRs occurred and only 10 (2.6%) decided to skip doses as ADRs were encountered. Based on chart review, treatment was changed for 78 (20.3%) patients and from which 79% were due to documented ADRs (p = 0.00). Among them, CNS symptoms (27.4%) and anemia (16.1%) were responsible for the majority of changes. Around four percent of patients were non-adherent to ART. Non-adhered participants and those on treatment changes were not statistically associated with self-reported ADRs. Only unemployment status (AOR = 1.76 (1.15 - 2.70), p = 0.01) and ADR duration of less than one month (AOR = 1.95 (1.28-2.98), p = 0.001) were significantly associated with self-reported adverse effects of three or more in the multivariate analysis.

**Conclusion:**

Self-reported ADRs to antiretroviral therapy are quite common. More of the reactions were of short lasting and their impact on adherence and treatment change were less likely. However, documented ADRs were the most prevalent reasons for ART switch. Moreover, the level of unemployment was a strong predictor of self-reported ADRs.

## Background

Worldwide, 34 million people are living with HIV from which 22.9 million (67.4%) are in the Sub-Saharan African countries. In 2010, there were an estimated 2.7 million new HIV infections and 1.8 million deaths due to AIDS [1]. According to literatures, the overall prevalence of HIV/AIDS in Ethiopia is 2.1% in 2010 [2]. The use of effective antiretroviral therapy (ART) has dramatically improved the pattern of morbidity and mortality among HIV infected patients, changing the disease pattern to a chronic manageable infection [3,4]. Highly active antiretroviral therapy (HAART) is known to increase CD4+ lymphocyte cell count which enhances immune system in HIV patients. The clinical benefits of HAART is characterized by increased survival and longevity in HIV patients [5]. However, these clinical benefits are associated with aversive adverse drug reactions (ADRs). Edward and his colleague defined ADR as an appreciably harmful or unpleasant reaction resulting from an intervention related to the use of a medicinal product, which predicts hazard from future administration and warrants prevention or specific treatment, or alteration of the dosage regimen, or withdrawal of the product [6].

Antiretrovirals, like most chronically administered drugs, are reported to have adverse reaction and particularly higher occurrences are seen at the beginning of ART [4,7,8]. Moreover, long term adverse effects such as lipodystrophy and neuromotor disorders may be encountered in latter stages of treatment [4]. Not only this, studies also showed that ADRs could be a source for new co-morbidities and hospital admission [9,10]. ADRs due to antiretrovirals can range from mild gastrointestinal disturbance [11] to serious adverse effects including hematological disorders [10], hepatotoxicity [12,13] and lactic acidosis [12]. A study done in Brazil among patients initiating ART in the first six month of therapy showed that at least one adverse reaction was reported by 92.2% of the participants while 56.2% reported four or more different reactions [8]. Singh *et al*. reported that 86% of patients had at least one ADR, of which, the most common observed was peripheral neuropathy [14]. A prospective observational study by Nagpal et al. reported that about 90% of patients experienced ADR [11]. In this study, non-compliance due to ADRs was observed in 28.9% of patients. Clinical benefits from ART can only be achieved with strict adherence and life-long treatment [3,4]. However, ADRs are considered the most limiting factor that compromises patient compliance and adherence. A number of authors reported ADRs as reasons for non-adherence [4,11,15-17]. Moreover, meta-analysis and review studies reported that non-adherence to treatment is one of the factors that leads to treatment failure and poor prognosis [12,18]. ADRs leave fewer options to clinicians and practitioners and compromise antiretroviral drug efficacy particularly if the intention is to withdraw and substitute the offending agent for the other. Furthermore, substitution is difficult in resource limited settings. Overall, ADRs become a concern and public health problem particularly in developing nations as adequate drug toxicity monitoring and reporting schemes barely existed. Lack of ADR monitoring and reporting system underestimates the burden of ART associated ADRs. Therefore, utilizing self-reported ADR studies could be one way of addressing such gaps. The aim of this study was thus, to assess the most common self-reported ADRs, determine associated factors and assess impacts of ADRs on treatment at ART clinic of Gondar University Hospital (GUH).

## Methods

Cross–sectional study was conducted at Gondar University Hospital (GUH) which is located in Gondar town of the Amhara regional state, 738 km North-west of Addis Ababa. GUH is the first teaching and referral hospital for the region. The hospital opened the area’s first ART clinic and began offering ART services in March 2002. At the time of the study period, GUH ART clinic was providing ART service to 6,163 HIV/AIDS patients. The study was carried out in ART clinic of GUH over three months period from April 1 to June 30, 2012. The study population was comprised of 4600 adult patients (18 years and above) who were active and on follow up at the ART clinic. Briefly, recruitment criteria were documented HIV positive status, at least 18 years of age, only those who had started only ART. Patients who were taking other medications along with ART, short-course ART for the sole purposes of prevention of mother-to-child transmission and post-exposure prophylaxis were excluded from the study.

Patients receiving ART treatment at GUH ART clinic were the sampling population for the study. The sample size was calculated based on a single proportion formula. The following parameters were used to calculate the sample size: total population of adult patients on ART 4600, proportion of patients who report at least one adverse reaction to ARTs 50% and 95% confidence interval with a marginal error of 5%. Additional 10% allowance for refusal to participate in the study was considered, which resulted in a total sample of 384. Using systematic random sampling method, patients were selected from the waiting room of ART clinic, on the day of their visit to refill ART medications. For data collection, a semi-structured interview questionnaire was used (Additional file 1). The questionnaire was first prepared in English, and then translated into Amharic. The questionnaire consisted of a list of common ART related symptoms, which were identified based on previously published studies [17,18] and pretested questionnaire. Before interviewing, patients were briefed about the definition pertaining to adverse drug reactions to ARTs. They were also asked if they had received any counseling about ADRs previously from physicians, pharmacists and nurses. In this study, adverse drug reaction refers to any undesirable symptoms reported by patients which they perceived it as a result of ART use. Respondents were asked whether they had experienced any of the range of options from the list. Additionally, patients were asked to report any other ADRs encountered during the course of therapy. In such a way, the outcome of interest was the number of adverse drug reactions which had occurred at least once since they had initiated ART.

Besides, socio-demographic, general health condition of the patient, questions related to impacts of self-reported ADRs on the treatment, social and psychological components were included. Patient medication chart review was also employed to extract information related to ART medications, ART treatment change, reasons for treatment change (documented ADRs, treatment failure, co-morbidity), baseline and current laboratory values. Recent self-reported antiretroviral medication adherence was assessed based on patients’ report on missed doses of ART medications over the past three-days.

The textual data obtained were sifted, organized and coded; and then entered and analyzed using SPSS statistical software version 20. Descriptive analysis of participants and self-reported adverse drug reactions were carried out. Logistic regression was employed for bivariate and multivariate analysis. Variables with a p value of less than 0.2 were fitted to the final model. Median number of self-reported ADRs was considered as the cutoff point. Patients who reported three or more types of reactions were compared to those who reported fewer. The strength of associations between self-reported adverse drug reactions and selected variables was estimated by the odds ratio with a 95% confidence interval. Chi-square test was also carried out to measure the association of selected self-reported ADRs with those factors affecting ART treatment. Statistical associations were considered significant at p < 0.05.

The following operational definitions were used:

• **Non-adherence** – failed to take >95% or missing dose of the antiretroviral drugs prescribed in the last three days prior to the interview

• **Healthy –** asymptomatic, physical activity not affected and not confined to bed at the start of treatment

• **Mild illness** – mild symptoms, physical activity not affected and not confined to bed at the start of treatment

• **Severe illness** – recurrent illness with wide range of infections, frequent confinement to bed, marked physical activity limitation and/or hospitalized due to prevailing conditions.

### Ethical consideration

The study was approved by the ethical review committee of School of Pharmacy, College of Medicine and Health Sciences, University of Gondar. Written informed consent was obtained from the respondents for the interview. Besides, confidentiality of the information was strictly maintained during data collection and data analysis process. Each interview questionnaire was assigned a study identification number. Respondents were also informed that their information would be used anonymously.

## Result

A total of 384 participants were enrolled in the study. Descriptive socio-demographic variables indicated that 85.1% of the participants were below 40 years old, 66.9% were females, 55.2% were married and 38.8% were unemployed. Most study participants had some education, primary and above (74.7%) and 90.1% were from urban dwellings. With regard to clinical status, mean level of CD4 cells/mm at the initiation of ART was 165.68 ± 93.8 while at the time of data collection was 376.7 ± 181.3. The majority of participants (52.9%) CD4 level was over 350. Participants had received ART for a mean of 42.3 (±24.1) months of treatment duration and had most recently been on ART triple regimen consisted of AZT/3TC/NVP (40.1%). Overall, only 3.6% of the participants taking ART medications indicated that they had skipped pills in the prior three days to result in less than 95% adherence. Initially, most patients were mild to severely ill. However, after the start of ART medication, most respondents’ health status was improved in terms of body weight and CD4 level. In the contrary, social interaction of most participants was not changed (Table 1).

**Table 1:** Descriptive analysis of socio-demographic, clinical variables and health status of the participants, ART clinic, Gondar University Hospital, June 2012

**Variables**	**n(%), N = 384**
**Socio-demographic**	
Education ( primary and above)	287(74.7)
Work status (unemployed)	149 (38.8)
Age (≤40 years)	327(85.2)
Gender (female)	257(66.9)
Marital status ( married)	212 (55.2)
Residency (urban)	346 (90.1)
**Clinical variables**	
Duration of ART ( ≤4 years)	155(40.4)
Current CD4 level ( cells/mm^3^ )	
< 200	47(12.2)
200-350	134(34.9)
>350	203(52.9)
**Current ART regimens (triple)**	
AZT/3TC/NVP	154 (40.1)
TDF/3TC/NVP	50 (13.0)
AZT/3TC/EFV	48(12.5)
D4T/3TC/NVP	44(11.5)
TDF/3TC/EFZ	42 (10.9)
D4T/3TC/EFZ	23(6.0)
Others	23(6.0)
Non adherence	14(3.6)
**General profiles**	
General health at the start of treatment	
Healthy	50(13.0)
Mild to severely ill	334 (87.0)
**CD**_**4 **_**count after treatment**	
Increased	345 (89.8)
Decreased	9(2.3)
No change	30(7.8)
**Health status after ART start**	
Improved	344(89.6)
Not improved	40(10.4)
**Body weight**	
Increased	323(84.1)
Decreased	23(6.0)
No change	38(10.0)
**Social interaction of patients after ART start**	
Increased	81(21.1)
Decreased	10(2.6)
No change	293(76.3)

Based on the chart review, one out of five participants had changed their first ART because of ADRs (79%), treatment failure (13%), tuberculosis co-morbidity (5%) and pregnancy (3%). There were statistically significant association with these documented ADRs (p = 0.00). Central nervous system symptoms, anemia and peripheral neuropathy were among the major documented ADRs responsible for treatment change (Table 2).

**Table 2:** Distribution of major reasons and documented ADRs responsible for treatment change (based on chart review), ART clinic, Gondar University Hospital, June 2012

**Variables**	n (%)^*^
**Reasons for treatment change (N = 78)**	
Due to ADRs	62 (79.0)^**^
Due to treatment failure	10 (13.0)
Due to TB co-morbidity	4 (5.0)
Due to pregnancy	2 (3.0)
**Documented ADRs responsible for treatment change (N = 62)**	
CNS symptoms	**17 (27.4)**
Anemia	10 (16.1)
Peripheral neuropathy	9(14.5)
Skin rash	8 (12.9)
Lipodystrophy	7(11.3)
Weight loss	4(6.5)
Stomach ache	3 (4.8)
Hepatotoxicity	3 (4.8)
Dental illness	1 (1.6)

^*^Percent frequency out of the total number of patients (reasons for treatment change, N = 78; ADRs responsible for treatment change, N = 62). The most frequent reasons are highlighted in bold type.

^**^95% CI, 0.68-0.88; p =0.00.

At least one ADR was reported by 345 (89.8%) patients due to the use of ART and the mean number of ADRs reported was 3.7 (±0.2). Most patients (94.6%) stated that they had received counseling regarding adverse reaction to ART from physicians, pharmacists or nurses before initiating and in the course of ART therapy. The most common self-reported adverse drug reactions were nausea (56.5%), headache (54.9%) and fever (40.9%) (Table 3).

**Table 3:** Most common self-reported adverse drug reactions of antiretroviral therapy, ART clinic, Gondar University Hospital, June 2012

**Self-reported adverse drug reactions**	n (%)^*^, N = 384
Nausea	217 (56.5)
Headache	211 (54.9)
Fever	157 (40.9)
Vomiting	147 (38.3)
Lethargy/fatigue	131(34.1)
Loss of appetite	130 (34)
Insomnia	102 (26.6)
Depression/stress	99 (25.8)
Skin rash	85 (22.1)
Night mare	72 (18.8)
Diarrhea	41 (10.7)
Oral ulceration/dry mouth	35 (9.1)
Anxiety	23 (6.3)
Others**	10 (2.6)

*number of frequency and percent proportions, total number of participants (N = 384).

**includes tingling in hands or feet, anemia.

Table 4 present results of the logistic regression analysis. In the bivariate analysis, unemployed status, urban residents, ART duration of more than four years, non-adhered patients and ADR duration of less than one month reported a high proportion of three or more different types of ADRs ( p < 0.2). Among the variables assessed for further analysis, unemployment status (AOR = 1.76(1.15-2.70), p = 0.01) and ADR duration of less than one month (AOR = 1.95 (1.28-2.98), p = 0.001) were significantly associated with self-reported adverse effects of three or more in the multivariate analysis.

**Table 4:** Logistic regression analysis of selected variables and adverse drug reactions to ART, ART clinic, Gondar University Hospital, June 2012

**Variables**	Total (N)^*^	Self-reported ADRs^** ^**(≥3), n = 229**	OR (95% CI)^***^	**P value**	**AOR( 95% CI)**^**†**^
**Age**					
≤ 40 years	327	192(58.7)	1		
>40 years	57	37(64.9)	1.28(0.69, 2.38)	0.44	
**Gender**					
Female	257	152(59.1)	1		
Male	127	77(60.6)	1.06 (0.69, 1.64)	0.78	
**Education**					
Illiterate	97	58(59.8)	1		
≥primary	287	171(59.6)	0.92 (0.58, 1.46)	0.72	
**Work status**					
Employed	149	78(52.3)	1		1
Non-employed	235	151(64.2)	1.82 (1.17, 2.84)	<0.01	1.76 (1.15, 2.70)^a^
**Marital status**					
Married	212	121(57.1)	1		
Non- married	172	108(62.8)	1.16 (0.75, 1.82)	0.50	
**Residency**					
Urban	346	211(61.0)	1		
Rural	38	18(43.4)	0.58(0.28, 1.18)	0. 14	
**Duration of ART**					
≤4 years	155	85(54.8)	1		
>4 years	229	144(62.9)	1.39 (0.92, 2.21)	0.11	
**ART treatment change**					
Yes	78	42(53.8)	1		
No	306	187(61.1)	1.35(0.82,2.22)	0.24	
**Current CD4 level**					
≤350	181	107(59.1)	0.96(0.64, 1.44)	0.85	
>350	203	122(60.1)	1		
**Current ART regimen**					
AZT backbone	204	122(59.8)	1.01(0.67, 1.53)	0.94	
Non-AZT backbone	180	107(59.4)	1		
**Adherence**					
Yes	370	223(60.3)	1		
No	14	6(42.8)	0.50(0.17, 1.45)	0.2	
**ADR duration (self-reported)**					
≤1 month	212	140(66.0)	1.81(1.20, 2.74)		1.95(1.28,2.98)^b^
>1 month	172	89(51.7)	1	<0.01	1

^*^Percent frequency that refers to the total number of patients (N = 384). ^**^Number and proportion of patients’ self-reporting 3 or more adverse drug reactions. ^***^Values represent odds ratio (OR) at 95% confidence interval. ^†^Values represent adjusted odds ratio (AOR) at 95% confidence interval, ^a^p = 0.01, ^b^p = 0.001.

Results of the impacts of self-reported ADRs showed that only 10 (2.6%) patients decided to skip doses as ADRs were encountered. But, none of the self-reported ADRs were statistically associated with such dose skipping trend. As a measure of impact of ADRs on patients’ perception regarding treatment, 114 (31.0%) of participants reported that they considered antiretroviral therapy to be unsuccessful due to the ADRs that they encountered. Moreover, it was found that such impacts of reported ADRs showed significant association with self-reported ADRs such as fever, vomiting, loss of appetite, diarrhea, insomnia, lethargy, dry mouth and depression. Similarly, various types of reported ADRs were significantly associated with the responses for questions linked with patients’ social interaction (Table 5).

**Table 5:** Impacts of self-reported ADRs on perception of treatment and social interaction, ART clinic, Gondar University Hospital, June 2012

**Variables**	**n (%)**	**Association of variables with specific ADRs (chi-square or fisher test)**										
		**Nausea**	**Headache**	**Fever**	**Vomiting**	**Lethargy**	**Loss of appetite**	**Insomnia**	**Depression**	**Night mare**	**Diarrhea**	**OU/DM**
**Impacts on patients perception on treatment patients who:**												
Decided to skip medication due to ADRs^*^	10 (2.6)	0.54	0.51	0.35	0.55	0.26	0.48	0.20	0.21	0.42	0.78	0.26
Consider ART unsuccessful due to ADRs^**^	114 (31.0)	1.43	0.10	7.42^b^	5.00^a^	29.53^c^	25.93 ^c^	14.47 ^c^	24.43^c^	1.43	13.63^c^	13.70^c^
**Impacts of ADR on patients’ social interaction patients who:**												
Perceive that people avoid them due to observed ADRs^**^	21 (32.9)	5.68 ^a^	0.76	11.97^c^	12.7^c^	28.13^c^	26.67 ^c^	18.28 ^c^	23.15^c^	3.19^a^	8.45^b^	14.94^c^
Tend to reduce/avoid social interaction because of ADRs^**^	119 (32.2)	2.11	0.26	9.87 c	7.96^b^	33.20	26.67^c^	20.88 _c_	3.40^c^	1.87	8.45^b^	6.70 ^c^
Perceive family members avoid them when ADRs occurred ^**^	57 (15.4)	0.99	3.35^a^	2.19	0.06	0.08	1.01	0.45	3.40^a^	0.03	0.99	6.70^b^
Talk to family members about ADRs as it appears^**^	296 (80.0)	14.32^c^	10.24^c^	5.92 ^b^	7.73^b^	4.34^a^	2.84	0.61	2.21	0.31	0.01	0.62

n = 384. ^*^Fishers exact test, ^**^chi-square test was used to assess associations. ^a^significant at a level of p < 0.05, ^b^significant at a level of p < 0.01, ^c^significant at a level of p < 0.001. OU/DM, oral ulceration or dry mouth.

## Discussion

This study was aimed at describing the commonly perceived ADRs, characterizing the impact on patients taking ART and associated factors with self-reported ADRs at ART clinic of Gondar University Hospital. Our findings indicated a high proportion of patients (89.8%) reported at least one adverse drug reactions because of antiretroviral therapy since they began ART. This finding is closely consistent with works of other studies that showed a high proportion of patients on ART reported at least one ADR to be as high as 86%-94% of the patients [8,11,14,19]. Almost, all these studies used a similar study design. This finding is relatively higher than the reports by Luma et al. [20] in Cameron and de Padua et al. [4] in Brazil. Our study assessed adverse reactions which were merely of patient self-report and thus, those symptoms perceived as adverse drug reactions to ARTs might be confounded with symptoms due to HIV. This is more evident as most patients reported fever as one of their ADR which is more likely to HIV and concurrent infections. In such occasions, this might lead to an overestimation of this outcome. On the other hand, this difference might be due to differences in the study design and lack of standardized definition of ADR among the different studies. Moreover, genetic and ethnic susceptibilities to ADR to a particular drug might also explain the difference.

In this study, the most common reported ADRs were from gastrointestinal symptoms; among which nausea was highly prevalent constituting more than half of all ADRs. In agreement with previous studies, gastrointestinal complaints were the reactions most commonly encountered [4,8,11]. In a Brazilian study of 406 patients, the most frequently reported adverse drug reaction was nausea where the occurrence was as high as 51.2% [8]. In the contrary, Singh et al. [14] and Luma et al. [20] reported that about a fifth of patients on ART developed peripheral neuropathy. Yet, a self-report by Melangu [19] in Pretoria, South Africa showed that sexual problems were the highest adverse effects reported. Few studies try to look the association between socio-demographic variables and adverse drug reactions, our study showed no difference in reported ADRs between age and sex differences. This finding is consistent with Eluwa et al. [7]. In this study, only unemployment status and ADR duration of less than one month were independently associated with self-reported ADRs of three or more. On top of the adverse drug reactions to ARTs, the level of unemployment in our study is a cause for concern because it results in a lot of psychosocial problems. Around one third of patients reported depression, stress and anxiety among the reported ADRs. Findings showed that unemployment is one of the causes of mental health problems [21,22]. We deduced that more of the reactions were of short lasting and their impact on adherence and treatment change were less likely. However, patient chart review showed that switching therapy was identified as major intervention used for the management of adverse reactions to ART in this population. Of those patients who had their ART regimen switched, 79% ascribed the change in regimen to the occurrences of adverse reactions due to ART. This is higher than the findings in 2007 in Brazil where ADR was found to account for 56.1% of all switches [4]. Unlike self-reported ADRs, the most documented reactions were more serious events like CNS symptoms, anemia and peripheral neuropathy. The patients’ perception of adverse reactions would potentially attribute to non-adherence to medications. But this study showed no difference in reported ADRs between the adhered and non-adhered population. We observed that 3.6% of patients were non-adherent to ART. This result is lower than that of the report by Nagpal et al. who reported non-adherence in 28.9% of Indian patients due to ADRs to ART (11). Other reports also showed non-adherence rate of 13%-21% [15,16,23]. The differences can be attributed to variation in the adherence assessment method.

Only 2.6% of subjects skipped medications because of perceived ADRs. Different studies mentioned various reasons for missing doses; forgetting, being away from home and being busy were the most common [16,24]. The lower rate of missing doses indicates the strength of patient education system and competent follow up by the health practitioners. However, perceived medication side effects and ADRs influenced patients’ perception on ART. Patients who considered ART as unsuccessful were significantly associated with the common ADRs including fever, GIT side effects, insomnia, lethargy and depression. Furthermore, these medication ADRs demonstrated significant influence on patient’s social interaction behavior. For instance, 32.9% of the study subjects perceive that people would avoid them when ADRs encountered and some 32.2% tend to reduce social interaction. On contrary to these, most of the study groups (80.0%) would love to share their feeling about the ADRs with their family members. Specifically, the result indicated a significant family interaction of patients when ADRs such as fever, headache, nausea, vomiting and lethargy had occurred.

This study has a number of limitations. The ADRs are self-report and there were no further investigation of laboratory tests and other diagnostic tests for ruling out for other possible causes which could lead to over-representation of ADRs. On the other hand, it might lead to under-estimation of ADRs which might have been detected clinically. Besides, the reporting could be influenced by the patient’s ability to memorize events related to adherence assessment and the duration of time ADRs lasted.

## Conclusion

Our study showed that perceived adverse drug reactions due to antiretroviral therapy are very frequent and prevalent in the studied population. More of the reactions were of short lasting and their impact on adherence and treatment change were less likely. Although treatment change and self-reported ADRs were failed to show statistical significance, documented ADRs were the most frequent reasons for ART switch. Unlike self-reported ADRs, documented ADRs were serious events. Moreover, the level of unemployment was a strong predictor of self-reported ADRs. Self-reported ADRs have a number of impacts on the patients’ perception to ART and drug taking behavior. Specific self-reported ADRs showed their influences on the patients’ perception to antiretroviral therapy and social interactions. Particularly, self-reported ADRs forced the patients to consider the antiretroviral therapy as if it was unsuccessful. In addition, self-reported ADRs demonstrated their potential threat to the patients social interaction as the patients considered people avoid them due to ADRs. Therefore, clinicians, caregivers and the patient himself/herself should actively collaborate for the better ART outcome because ADRs are a potential threat to the effectiveness of ART. ADR reporting, monitoring and management should further be strengthened to enhance better ART service. Patient education on ART associated ADRs should be an integral part of HIV care so as to facilitate reporting and management.

## Competing interests

The authors declare that they have no competing interests.

## Authors’ contributions

Data were collected by WTT and WHT. WTT, ABM and YTT contributed in statistical analysis. All authors were involved in manuscript write-up and correction. The final version of the submitted manuscript was approved by all authors.

## Pre-publication history

The pre-publication history for this paper can be accessed here:

http://www.biomedcentral.com/2050-6511/15/32/prepub

## Supplementary Material

Additional file 1: Annex 1 Study questioner used to collect variables and self-reported adverse drug reactions at ART clinic, Gondar University Hospital, April 2012. Click here for file
