# Supplementary material for: Self-reported adverse drug reactions and their influence on highly active antiretroviral therapy in HIV infected patients: a cross sectional study
Source: BMC Pharmacol Toxicol. 2014 Jun 23;15:32. doi: 10.1186/2050-6511-15-32 (PMC4082293; doi:10.1186/2050-6511-15-32)
Supplement: Additional file 1: Annex 1 — Study questioner used to collect variables and self-reported adverse drug reactions at ART clinic, Gondar University Hospital, April 2012. [file 2050-6511-15-32-S1.docx]

**Annex1:** **Study questioner used to collect variables and self-reported adverse drug reactions at ART clinic, Gondar University Hospital, April 2012.**

This interview questioner is prepared to interview and gather information related to ART medication, perceived ADRs and their impact on treatment.

**Part I: socio-demographic**

1. Age _________
2. Gender: _________
3. Education
4. Illiterate C. Secondary
5. Primary D. College
6. Marital status
7. Married C. Widowed
8. Single D. Divorced
9. Residency
10. Urban
11. Rural
12. Work status
13. Unemployed
14. Employed

**Part II Health condition**

1. When did you start ART ______Month _______Year _______ (from medication record)
2. ART treatment regimen _________________________ (from medication record)
3. Starting CD4 level ____________ Current CD4 ____________ (from medication record)
4. Baseline Body weight _________ current body weight _________ (from medication record)
5. How was your health condition before the start of ART medications?
6. Healthy
7. Mild to severely ill
8. How is your health condition after the start of ART medication?
9. Improved
10. Not improved
11. How is your social interaction after ART start?
12. Increased
13. Decreased
14. No change
15. Have you received any advice about ADRs from health professionals?
16. Yes
17. No
18. Did you encounter ADR during your course of ART therapy?
19. Yes
20. No
21. If your answer is ‘Yes’ to question number 9, which adverse drug reactions were encountered during your course of therapy
    1. Headache
    2. Nausea
    3. Skin rash
    4. Tingling of feet /hands
    5. Lethargy/Fatigue
    6. Night mare
    7. Depression
    8. Anemia
22. Any other adverse drug reactions you encountered since you have started antiretroviral therapy ______

**Part III Impact of ADRs on the treatment and on the patients**

1. Over the last 3 days did you miss treatment regimens?
2. Yes
3. No
4. How many tablets did you miss in the last 3 days? ___________
5. Have you had treatment changes? (from medication record)
6. What was the reason for treatment change? (from medication record)
7. Due to the ADRs, have you ever decided to skip medications?
8. Yes
9. No
10. Do you consider ART unsuccessful due to ADRs you encountered?
11. Yes
12. No
13. Do you feel people avoided you due to the ADRs that appear on course of therapy?
14. Yes
15. No
16. Do feel stigmatized just after the start of your ART regimen by your family members?
17. Yes
18. No
19. Have you ever discussed with your family or close friends about ART medication ADRs you encountered?
20. Yes
21. No
